# Hippocampal network activity changes during early epileptogenesis predict subsequent epilepsy

**DOI:** 10.1002/epi.70386

**Published:** 2026-07-18

**Authors:** Michael Strüber, Tristan Manfred Stöber, Vanessa Schütz, Lara Costard, Valentin Neubert, Sebastian Bauer, Parsa Gharavi, Julika Pitsch, Nico Melzer, Adam Strzelczyk, Jochen Triesch, Kai Siebenbrodt, Felix Rosenow, Ricardo Kienitz

**Affiliations:** ^1^ Epilepsy Center Frankfurt Rhine‐Main, Department of Neurology, University Hospital Frankfurt Goethe University Frankfurt Frankfurt am Main Germany; ^2^ LOEWE Center for Personalized Translational Epilepsy Research (CePTER) Goethe University Frankfurt Frankfurt am Main Germany; ^3^ Cooperative Brain Imaging Center (CoBIC) University Hospital Frankfurt Frankfurt am Main Germany; ^4^ Institute of Computer Science and Campus Institute Data Science University Göttingen Göttingen Germany; ^5^ Circulant Labs Bensheim Germany; ^6^ Department of Neurology Philipps University Marburg Germany; ^7^ Oscar Langendorff Institute of Physiology University of Rostock Rostock Germany; ^8^ AISA Solutions Inc. Cleveland Ohio USA; ^9^ Department of Epileptology University Hospital Bonn Bonn Germany; ^10^ Department of Neurology, Medical Faculty and University Hospital Düsseldorf Heinrich Heine University of Düsseldorf Düsseldorf Germany; ^11^ Frankfurt Institute for Advanced Studies Frankfurt am Main Germany

**Keywords:** aperiodic activity, epilepsy, excitation/inhibition ratio, neuronal networks, oscillations

## Abstract

**Objective:**

Despite decades of research, the circuit mechanisms that underlie focal epileptogenesis remain incompletely understood. In this study, we aimed to characterize the changes in hippocampal network activity induced by an epileptogenic insult.

**Methods:**

In rats, prolonged electrical perforant pathway stimulation leads in a high percentage of animals to the development of temporal lobe epilepsy. However, a subset of animals remained apparently non‐epileptic for unknown reasons. We monitored alterations of neuronal activity by chronically recording the local field potential (LFP) from the hippocampal dentate gyrus before, during, and after the potentially epileptogenic insult and quantifying the time‐dependent changes in the periodic and the aperiodic components of the LFP.

**Results:**

It is intriguing that animals with epilepsy identified by subsequent spontaneous epileptic seizures were characterized by a transient increase in the aperiodic exponent, suggesting a shift toward a reduced local excitation‐to‐inhibition (E/I) ratio during the first days after the perforant path stimulation. Furthermore, these animals developed a strong impairment of theta oscillation prevalence and regularity during early epileptogenesis. In contrast, resilient non‐epilepsy animals without spontaneous seizures showed neither this modulation in E/I ratio nor a corruption of hippocampal theta activity. In fact, the increase in the aperiodic exponent on the first day after completion of the electrical stimulation paradigm could predict epileptogenesis with very high fidelity (area under the curve [AUC] 0.92) and correlated significantly with later seizure rate.

**Significance:**

The very early phase of epileptogenesis seems to be dominated by a relative dominance of inhibitory synaptic activity in affected circuits. This finding opens the opportunity to dissect mechanisms of epileptogenesis and to test the effectiveness of anti‐epileptogenesis treatment in very early disease stages by allowing identification of individuals at high risk. Furthermore, it might offer a potential explanation for the frequently observed failure of anti‐epileptogenesis drugs boosting γ‐aminobutyric acid (GABA)ergic inhibition.

**Key points**

- Electrically induced hippocampal epileptogenesis induces a reduced excitation/inhibition (E/I) ratio at a very early disease stage.
- Animals intrinsically resilient against epileptogenesis induction do not show this shift in E/I ratio.
- Hippocampal theta oscillations are strongly impaired early during epileptogenesis.
- Animals developing epilepsy after weeks can be already identified immediately after epileptogenesis induction by an early drop in E/I ratio.

## 1 INTRODUCTION

Temporal lobe epilepsy is a common neurological disorder, characterized, in part, by dysfunctional cortical circuits and epileptic seizures.^1^ The excessive and highly synchronized neuronal activity in epilepsy causes severe paroxysmal symptoms such as loss of consciousness, falls, involuntary body movements, or vegetative dysregulation with apnea or asystole in extreme cases. On the level of the brain networks, the exact mechanisms of seizure generation and the alterations underlying the propensity of neuronal networks to generate seizure activity spontaneously are only partially understood. Inhibitory interneurons are prominent regulators of neuronal activity and they play an important role for brain function in health and disease.^2^ Their inhibitory action can counterbalance excessive excitation in neuronal networks, but they can also exert a possibly pro‐epileptic synchronizing effect on their target cell population.^3^, ^4^ Thus, it is not surprising that data on their pro‐ or anti‐epileptic roles are ambiguous and sometimes contradictory.^5^

The changes occurring during the development of acquired focal epilepsy, the so‐called *epileptogenesis* period, are complex and may involve neuronal, glial, immune and blood–brain barrier (BBB)–related mechanisms.^1^, ^6^, ^7^, ^8^ However, essential for the strong propensity of brain tissue to generate seizures are alterations in neuronal networks, rendering them more excitable.^9^ One critical determinant of neuronal network excitability is the ratio of excitatory and inhibitory synaptic activity distributed in the circuit, the so‐called excitation/inhibition‐ (E/I‐) ratio.^10^ The time‐dependent changes of E/I ratio in neuronal networks during their epileptogenic conversion to hyperexcitable circuits have not been addressed.

Novel methods have been developed recently to estimate the amount of inhibition in neuronal networks by analysis of local field potential (LFP) recordings.^11^, ^12^ In this study, we monitored changes in hippocampal network excitability over the course of epileptogenesis in a toxin‐free model of electrically‐induced temporal lobe epilepsy in rats.

## 2 MATERIALS AND METHODS

### 2.1 Animals and electrode positioning

All animal experiments were conducted in accordance with national and institutional guidelines (EU directive 2010/63/EU; approved by the Regierungspräsidium Gießen and the Regierungspräsidium Darmstadt). Altogether 18 adult Sprague–Dawley male rats (weight 300–450 g) were housed individually to avoid damage of implants by littermates. Animals had free access to food and drinking water; a 12 h/12 h light/dark cycle was maintained. Six of the 18 included animals have already delivered data to a previous study.^13^

The surgical procedure has been described in detail previously.^13^, ^14^ Briefly, stimulation electrodes (0.125 mm in diameter) were implanted bilaterally in the angular bundle of the perforant path (coordinates: ±4.5 mm lateral to bregma, 0.5 mm rostral to lambda, variable depth). Furthermore, a recording electrode (0.25 mm in diameter) was implanted unilaterally in the left hippocampus (coordinates relative to bregma: 2 mm lateral, 3 mm caudal, variable depth). Over three consecutive days, electrical stimulation was applied over 30 min on the first 2 days and over 8 h on the third stimulation day. A more detailed description of the surgery and epileptogenesis induction protocol can be obtained from the Supporting Information; the electrical stimulation pattern is shown in Figure 1A.

**FIGURE 1:**
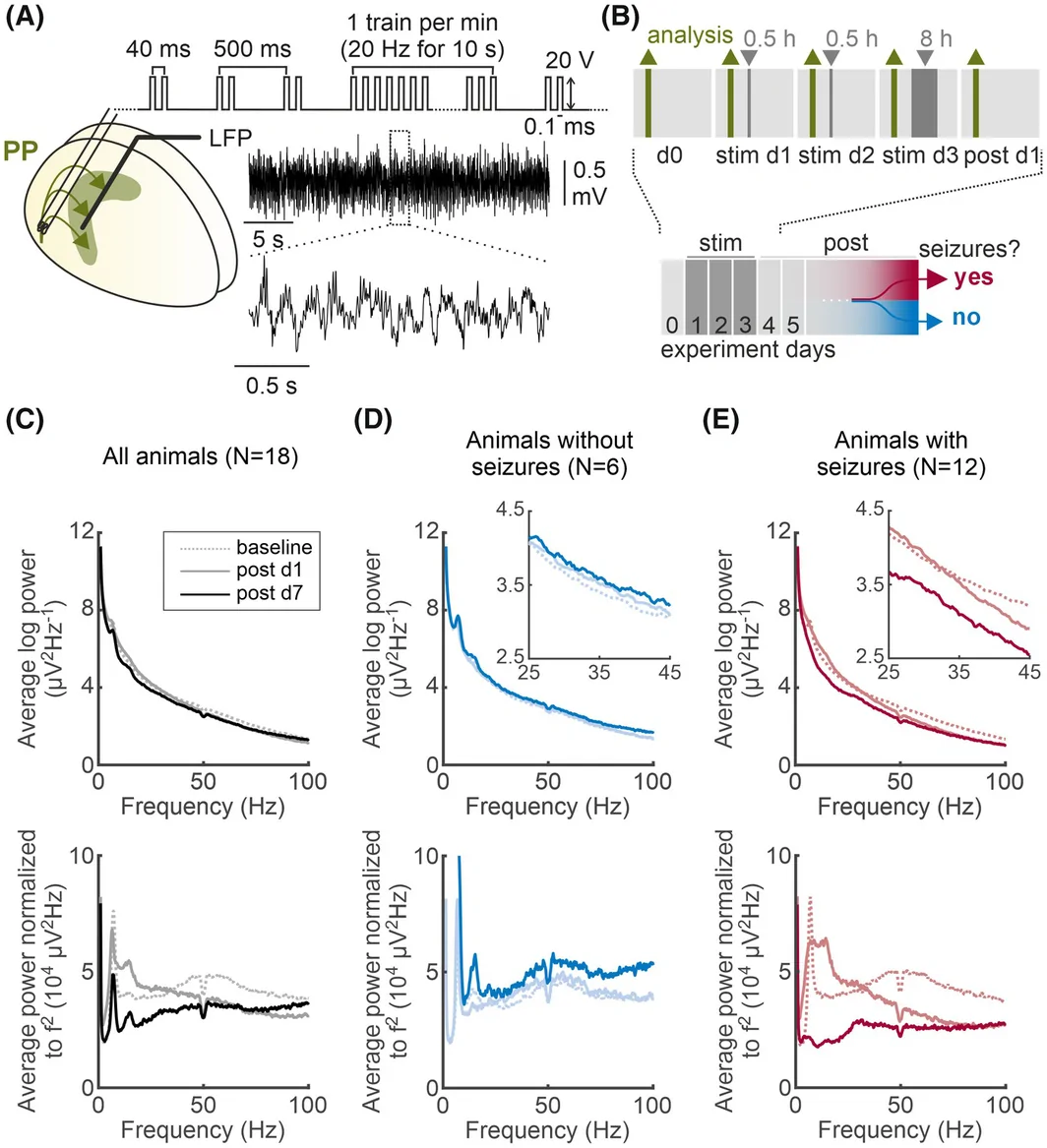
Electrically induced early epileptogenesis. (A) Experimental setup with bilateral perforant path (PP) stimulation and continuous local field potential (LFP) recordings from hippocampal dentate gyrus. Top, stimulation protocol; middle, representative recorded LFP; bottom, magnified episode with prevalent theta oscillations. (B) Time line of the experiment is schematically shown. On three stimulation days, the stimulation protocol shown in (A) is applied over 30 min on stim Days 1 and 2 and over 8 h on stim Day 3, starting at around noon on the respective day. Quantitative analysis of network activity is performed at 3–5 a.m. on the indicated days (green bars, top schematic). After completion of stimulation, most animals develop spontaneous epileptic seizures after ~21 days (epilepsy), whereas a minority remained free from spontaneous seizures (non‐epilepsy). (C–E) Power density analysis of the LFP recorded during baseline (light color, dashed line) and on the first (light color, continuous line) and seventh day (dark color) after stimulation averaged over all animals (C, black), over all non‐epilepsy animals (D, blue) and over all epilepsy animals (E, red). Top, power plotted after logarithmic transformation; bottom, power is frequency‐normalized by multiplying the power by the corresponding squared frequency (f^2^). Insets, focus on the frequency range 25–45 Hz. Note, specifically on post Day 1 the slope of logarithmic power spectrum is increased in epilepsy animals.

### 2.2 Analysis of electrophysiological data

All data analysis was performed in MATLAB (The Mathworks, version R2022a). To prevent the results from being biased by electrical artifacts, electroencephalography (EEG) snippets devoid of artifacts with a duration of 2 min were manually selected for further analysis. To characterize long‐term changes during the course of the experiment independent from daytime, for every analysis day, EEG snippets were isolated at 3, 4 and 5 a.m. and the respective results were averaged. For the analysis of the time course around the individual stimulation sessions with a higher temporal resolution, again three individual 2 min EEG snippets were manually selected at pre‐defined latencies from the end of the respective stimulation (i.e., 30 min, and 1, 2, 4, 8, and 18 h). As a baseline for every stimulation session, the EEG snippets of the respective stimulation day collected in the morning hours (3–5 a.m.) were used. Stimulation usually commenced at 11 a.m. to 1 p.m. of the respective day.

To remove 50 Hz power net artifacts, EEG signals were, in a first step, bandpass‐filtered using a power spectrum interpolation filter (*dft* filter of the fieldtrip toolbox^15^). Power spectra were obtained using the *mtspectrumc* function of the Chronux toolbox (http://chronux.org). Analysis of the aperiodic activity and determination of the aperiodic exponent were accomplished using the *fooof*‐algorithms of the fieldtrip toolbox^15^ with analysis over the frequency range 1–95 Hz. However, similar results have been obtained by performing the analysis over the frequency range 25–45 Hz.

### 2.3 Statistics

Statistical significance was tested using two‐tailed versions of the indicated tests. Distributions were tested for normality using Lilliefors‘test (MATLAB's *lillietest*). Whenever the normality hypothesis was rejected, a Wilcoxon rank‐sum test was used. Otherwise an unpaired two‐sample *t*‐test or a multiple comparison analysis of variance (ANOVA) was employed.

Values in the text are given as mean ± standard error of the mean (SEM), if not stated otherwise. To analyze correlations, Spearman rank analysis was used.

To investigate the influence of outcome (*epilepsy* vs *non‐epilepsy*) on the prevalence of significant theta oscillation activity, repeated‐measures ANOVA was used (MATLAB's *fitrm* and *ranova*). The *p*‐value given in the text describes the interaction between outcome and time (Figure 3B).

To estimate the fidelity of epileptogenesis prediction on the basis of the aperiodic exponent change upon perforant path stimulation in 18 animals, a linear discriminant classification model was used in a leave‐one‐out cross‐validation approach. In detail, we generated 18 training sets each consisting of 17 animals with a different animal left out in each classification round. In every round, a linear discriminant classification model using MATLAB‘s *fitcdiscr* function was trained on the training data and tested with the data of the animal left out in the respective round. Receiver operating characteristic (ROC) analysis (Figure 2F) was performed on the 18 prediction scores resulting from the cross‐validation procedure using MATLAB's *rocmetrics* function. No oversampling was performed due to only mild class imbalance (12 vs 6 observations).

**FIGURE 2:**
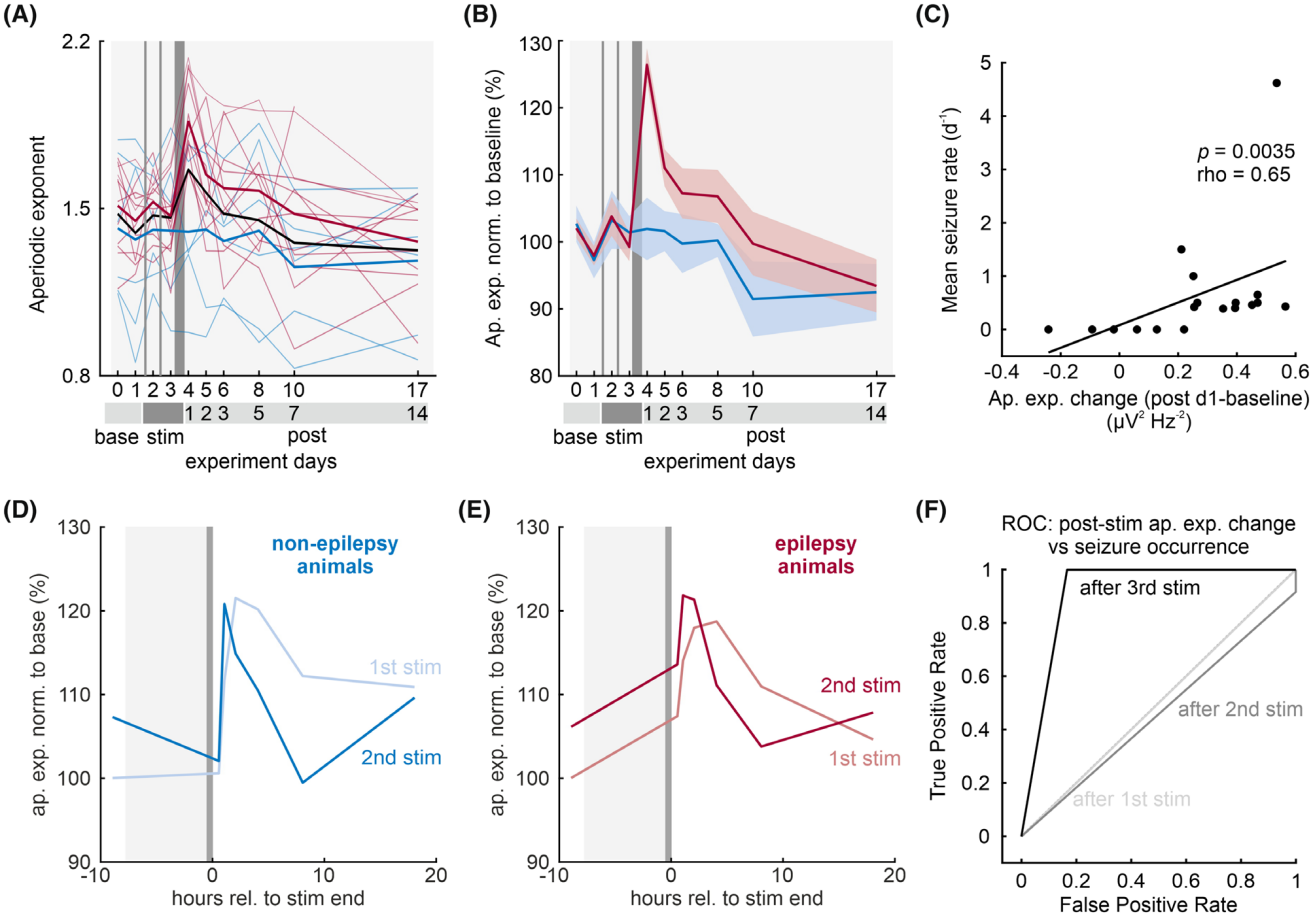
Transient reduction of excitation/inhibition (E/I) ratio during initial epileptogenesis. (A) Time course of the aperiodic exponent as a proxy for the local E/I ratio before, during, and after electrical stimulation over 3 days (dark gray vertical bars). Thin lines represent individual non‐epilepsy (blue, 6 animals) and epilepsy animals (red, 12 animals). Bold lines, average over all 18 animals (black), over non‐epilepsy (blue) or epilepsy (red) animals. Experiment Days 4–17 translate to post‐stimulation days 1–14. (B) Mean time course of the aperiodic exponent during the experiment in non‐epilepsy (blue) and epilepsy (red) animals. Lines, mean; shaded areas, standard error of the mean (SEM). Epilepsy but not non‐epilepsy animals show a transient increase of the aperiodic exponent lasting several days after completed stimulation (*p* = .014, repeated‐measures ANOVA). (C) Post‐stimulation increase of the aperiodic exponent correlates significantly with the chronic seizure rate. *p*, rho, Spearman rank correlation analysis. (D, E) After stimulation on Days 1 and 2, the aperiodic exponent on average increases for a period of a few hours in both non‐epilepsy (blue, D) and epilepsy (red, E) animals. (F) Receiver operating characteristic (ROC) analysis illustrating the predictive value of the aperiodic exponent change ~16 h after stimulation start for the classification of animals as non‐epilepsy or epilepsy (after first stim, area under the curve (AUC) 0.5; after second stim, AUC 0.46; after third stim, AUC 0.92).

## 3 RESULTS

In response to the long‐lasting electrical hippocampal stimulation over three consecutive days modeling electrically induced epileptogenesis (Figure 1B; Section 2), 12 of 18 included animals developed spontaneous seizures after a mean latency of 21.4 ± 3.1 days (*epilepsy* rats) and 6 showed no spontaneous electrographical seizures during the observation period (*non‐epilepsy* rats). Six of 18 animals showed acute symptomatic seizures defined as seizures occurring in the first 7 days after completion of stimulation^16^ (33% prevalence of acute symptomatic seizures in *epilepsy* and *non‐epilepsy* rats, respectively; see Figure S1). We characterized changes in hippocampal network activity induced by the perforant path stimulation by power spectrum density analysis (Figure 1C–E). Recent methodological advances suggested the inference of the ratio of excitatory and inhibitory synaptic activity in neuronal circuits from the 1/*f* ^χ^ relationship of aperiodic LFP signal power, with χ being coined the *aperiodic exponent*.^12^ Although higher ratios of aperiodic excitatory‐to‐inhibitory (or E/I) activity are reflected by flatter power spectra particularly for the sub‐gamma frequency range (≤40–50 Hz), lower E/I ratios are associated with more steeply declining power spectra.^11^, ^12^ Surprisingly, *epilepsy* animals, but not *non‐epilepsy* animals, showed more steeply declining power spectra in the frequency range of 25–45 Hz on the first day after stimulation compared to both baseline and 7 days after stimulation (Figure 1D,E insets).

To further investigate this finding, we determined for every animal the aperiodic exponent at multiple time points before, during, and after stimulation. On average, the aperiodic exponent seemed to increase strongly and for several days after the third, 8 h lasting stimulation but was reduced to baseline levels already on Day 7 after stimulation (Figure 2A, black line). When analyzing *epilepsy* and *non‐epilepsy* rats separately, a prominently elevated aperiodic exponent upon stimulation was found only in those rats developing spontaneous seizures during the following weeks (Figure 2A,B, *p* = .014, repeated measurements ANOVA). In support of this finding, we found a significant positive correlation between chronic seizure rate and the absolute increase in the aperiodic exponent on the first day after completion of the stimulation (Figure 2C). Next, we checked whether the shorter stimulations on Days 1 and 2 could induce shifts in the aperiodic exponent, too. We monitored the aperiodic exponent at a higher temporal resolution around the stimulation time points and found a substantial but only brief increase following the 30 min stimulation in both *epilepsy* and *non‐epilepsy* animals (Figure 2D,E; see also Figure S2). Thus, highly intense 30 min–lasting perforant pathway stimulation induces an increase in the aperiodic exponent for hours suggesting a brief shift in the E/I ratio toward lower values. However, long‐lasting stimulation over 8 h seems to induce a much more stable state of inhibitory dominance in animals developing chronic epilepsy. Finally, we performed a receiver operating characteristic (ROC) analysis of a linear discriminant classification model trained on the data of all 18 animals and found that the change in the aperiodic exponent after the third stimulation day but not after the first or the second stimulation day could predict subsequent development of epilepsy with high fidelity (AUC = 0.92; Figure 2F).

Strength of local synaptic excitation and inhibition in the dentate gyrus can be assessed by the field potential responses to paired‐pulse perforant path stimulation.^14^, ^17^, ^18^ In a subset of 10 animals, we analyzed the field excitatory postsynaptic potentials (fEPSPs) and population spikes in response to paired‐pulse perforant path stimulation during the epileptogenesis induction protocol (Figure S3). Pooling epilepsy and non‐epilepsy animals, we found on average both a systematic reduction of fEPSP slope as a proxy for afferent excitation strength and an increase of the paired‐pulse index (PPI) of population spike amplitudes indicative of a reduction in γ‐aminobutyric acid (GABA)ergic feedforward and feedback inhibition over the first 30 min of the stimulation protocol on all 3 days (Figure S3C). For the 8 h‐long stimulation on Day 3, the PPI was maximal at 2 h after stimulation onset before returning to lower values toward the stimulation end. Similar to previous findings,^14^ our data point to a stronger local GABAergic inhibition at the beginning of the 8 h stimulation on Day 3 as compared to baseline conditions at the beginning of the 30 min stimulation on Day 1 (Figure S3C, right). Although not being statistically significant (mean PPI beginning D1–19.5%, mean PPI beginning D3–42.9%, *p* = .306, unpaired *t*‐test), this might suggest boosting of inhibitory circuits by the preconditioning stimulation on Days 1 and 2. Of note, our analysis did not detect a systematic difference neither in amplitude or slope of fEPSPs nor in the size of population spikes between epilepsy and non‐epilepsy animals (Figure S3D; *p* = .187 for fEPSP amplitude, *p* = .761 for fEPSP slope, *p* = .334 for population spike amplitude, respectively; repeated‐measures ANOVA).

To further characterize hippocampal network function, we studied the properties of theta oscillations, the most prominent oscillatory pattern in the hippocampus strongly associated with animal locomotion.^19^ To avoid any behavior‐dependent bias, we selected LFP epochs with significant oscillatory activity in the theta frequency range (5–9 Hz) for further analysis (see Supporting Information Materials and Methods). The average theta power normalized to delta power and the fraction of LFP epochs showing significant theta activity were not different in *non‐epilepsy* and *epilepsy* animals during baseline conditions and after stimulation Days 1 and 2 (Figure 3A,B). However, after the final stimulation on Day 3, *epilepsy* animals showed significantly reduced normalized power and prevalence of hippocampal theta activity compared to *non‐epilepsy* rats (theta prevalence: *p* = .021, repeated‐measures ANOVA; Figure 3A,B). Next, we focused only on LFP epochs containing significant theta oscillatory activity (cf. Figure S4). To detect changes in the theta oscillation characteristics, we obtained mean theta cycle waveforms aligned to the oscillatory trough averaged over all *non‐epilepsy* and *epilepsy* animals (Figure 3C). During the course of early epileptogenesis, average theta cycles seemed to be horizontally stretched and vertically compressed in *epilepsy* rats. In order to dissect the underlying alterations in oscillation structure, we performed a cycle‐by‐cycle analysis of theta activity at different time points (Figure 3D,E). Theta cycle period showed a sudden increase upon completion of the stimulation pattern already on *post D1* in *epilepsy* but not *non‐epilepsy* animals. However, theta cycle amplitude was not altered by the stimulation protocol. Next, we probed stability of theta oscillations and studied the cycle‐to‐cycle variability in both period duration and amplitude of theta activity. For both, cycle amplitude and period duration a marked increase in the cycle‐to‐cycle variability became evident for *epilepsy* but not *non‐epilepsy* animals on Day 7 after stimulus completion (Figure 3D,E). Thus, early epileptogenesis after electrical perforant path stimulation is characterized by a substantially reduced regularity of theta oscillations. To test whether impairment of hippocampal theta activity results from strongly reduced locomotion in epilepsy animals, we made use of movement‐related variation in the telemetry signal quality and quantified the variance of signal dropout rate as a proxy for animal locomotion^20^, ^21^ (Figure 3F). Of note, this locomotion score indicated increased animal movement during dark phases 7 days after perforant path stimulation compared to baseline for both *epilepsy* and *non‐epilepsy* animals, suggesting that the impairment of hippocampal theta oscillations was not a mere consequence of reduced locomotion in *epilepsy* animals (Figure 3F).

**FIGURE 3:**
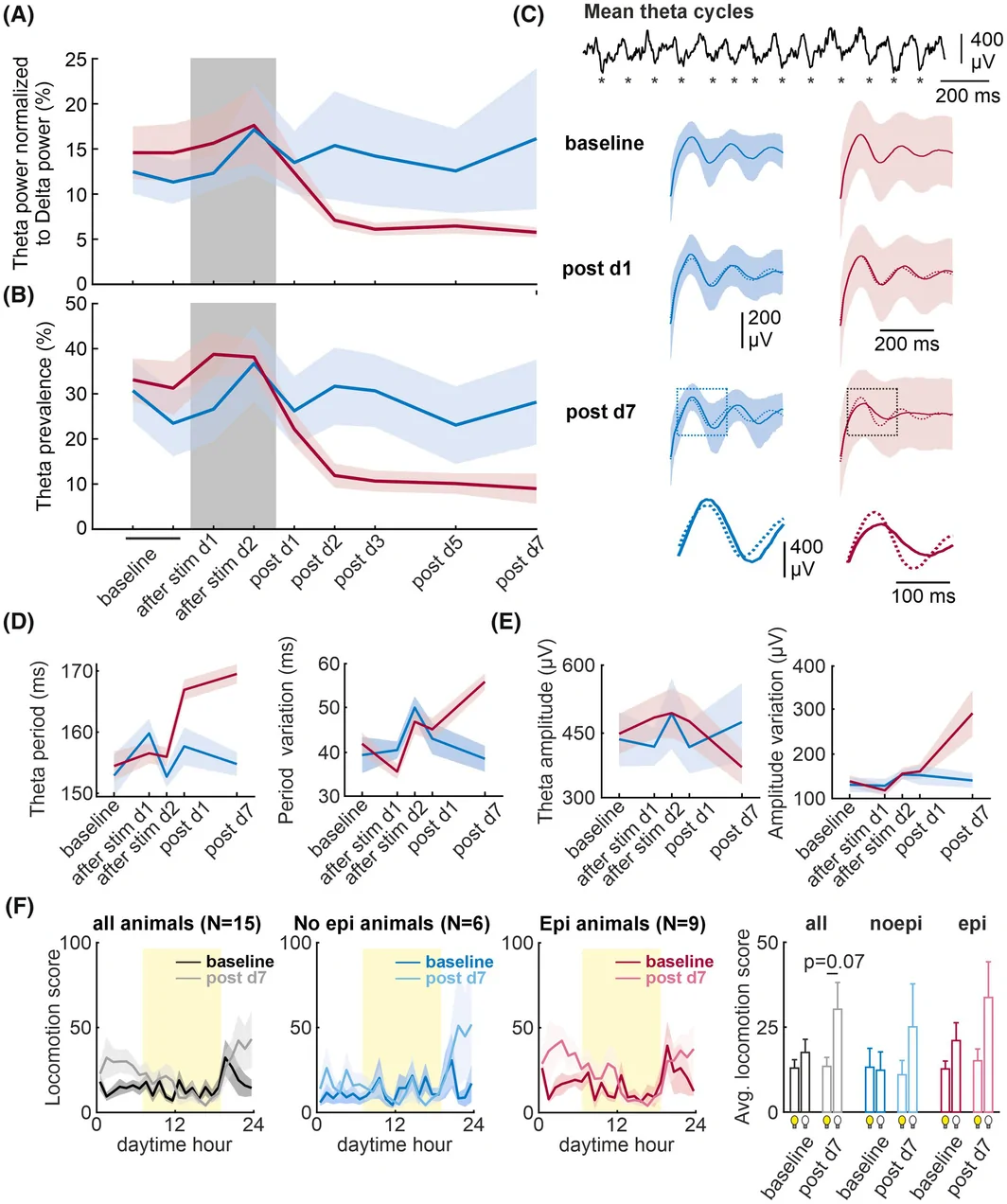
Reduced theta prevalence and regularity during early epileptogenesis. (A, B) Relative theta oscillation power normalized to delta oscillation power (A) and percentage of analyzed epochs classified as theta‐dominated (B) for non‐epilepsy (blue) and epilepsy (red) animals at different time points of the experiment. (C) Top, representative theta‐dominated hippocampal recording with theta troughs labeled by asterisks. Below, mean theta oscillation waveform aligned to theta troughs and averaged over all non‐epilepsy (left, blue) and epilepsy (right, red) animals at baseline and first and seventh days after stimulation. Continuous lines, average theta waveform; dashed lines, baseline waveform. Bottom, magnification of average theta waveforms indicated by dashed boxes. (D, E) Average theta cycle properties at different time points: Period duration (D, left); variation of period duration of consecutive cycles (D, right); amplitude (E, left); variation of amplitude of consecutive cycles (E, right). (F) Left, average locomotion score based on the telemetry signal dropout rate for all animals (black, left), the epilepsy animals (red, middle), and the non‐epilepsy animals (blue, right) determined during baseline and on the seventh day after perforant path stimulation. Rightmost, average locomotion score during the 12 h “light on”(yellow bulb) and the 12 h “dark” phases for the same experimental conditions. *p*, multiple comparison ANOVA test. Error bars, standard error of the mean (SEM). Lines, mean; shaded areas, standard deviation (A–C) or SEM (D–F).

In the hippocampal dentate gyrus, theta activity temporally structures excitatory and inhibitory synaptic activity with afferent excitation from the entorhinal cortex arriving at granule cells shortly before the theta trough with a mean theta phase angle of −39°.^22^ Indeed, when pooling all animals during baseline conditions and determining the aperiodic exponent at different theta phases, the aperiodic exponent showed a clear theta phase–dependent modulation with a minimum at −30° (Figure 4A), indicating maximal glutamatergic synaptic activity shortly before the theta trough as shown by Pernía‐Andrade and Jonas.^22^ To test epileptogenesis‐related alterations in the temporal structure of synaptic excitation and inhibition, we quantified the theta phase–dependent modulation of the aperiodic exponent at *post D1* and *post D7* in *epilepsy* and *non‐epilepsy* animals (Figure 4B). Although in the *non‐epilepsy* animals the aperiodic exponent displayed at all analyzed time points a clear minimum related to the theta trough, this phase modulation was inverted in *epilepsy* animals on *post D1* with a maximum shortly after the theta trough. On *post D7*, theta phase modulation of the aperiodic exponent returned to baseline levels with a minimum at the time point of theta troughs. Thus, the reduction in E/I ratio in early epileptogenesis suggested by a long‐term post‐stimulation increase in the aperiodic exponent seems to be most prominent during the theta trough when the afferent perforant path input has been shown to excite the dentate gyrus circuit.

**FIGURE 4:**
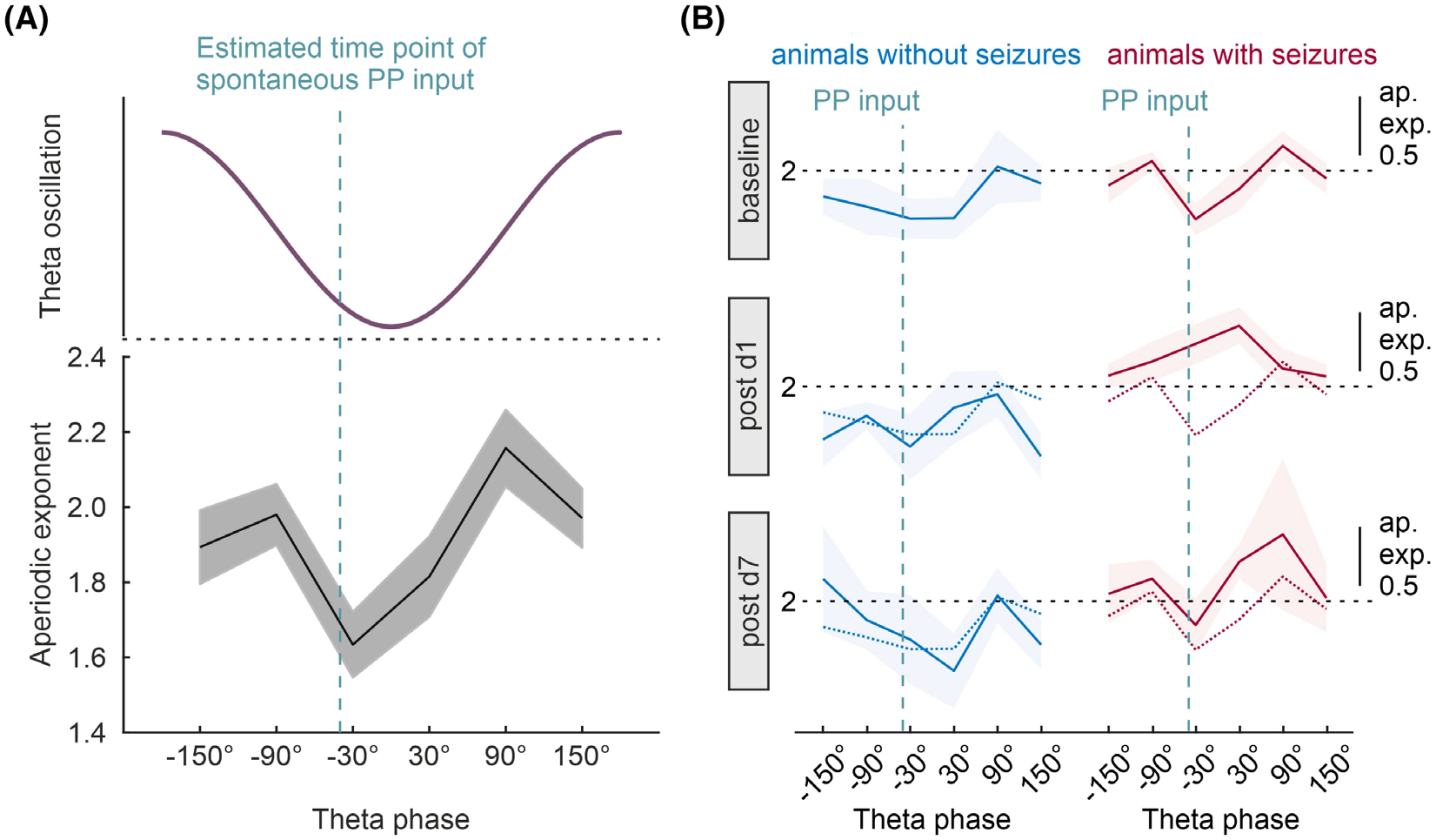
Epileptogenesis‐associated elevation of inhibitory dominance is theta phase dependent. (A) Theta phase‐dependent modulation of the aperiodic exponent with a minimum at −30° corresponding well to the phase of estimated maximal perforant path–mediated excitation of dentate gyrus granule cells^22^ (cyan broken line). Black line, average over all 18 animals during baseline; shaded area, standard error of the mean (SEM). (B) Evolution of the theta phase–dependent aperiodic exponent after perforant path stimulation in non‐epilepsy (blue, left) and epilepsy (red, right) animals. Continuous lines, average over all animals at the respective experimental day; broken lines, baseline; shaded areas, SEM.

## 4 DISCUSSION

The neuronal circuit mechanisms underlying the progressively developing propensity of epileptic networks to generate seizures are to date far from being fully understood. Synaptic inhibition provided by GABAergic interneurons is known to play an ambiguous role in the generation of seizures with both seizure‐suppressive and seizure‐igniting effects.^5^ Our results obtained in an electrically induced temporal lobe epilepsy rat model suggest a transiently increased relative abundance of inhibition immediately after application of an epileptogenic incident specifically in animals found to subsequently develop epilepsy (Figure 2). This transient post‐incidental putative dominance of circuit inhibition could actually predict the development of epilepsy with very high fidelity and might therefore be classified as a potential electrophysiological marker for early epileptogenesis.

Whether the observed change in E/I ratio is indeed due to elevated inhibitory synaptic activity, a reduced excitatory neurotransmission, or both is not clear. Furthermore, the cellular processes underlying the alteration in E/I ratio remain to be determined in future studies. The longer time scale of the E/I ratio shift toward inhibition lasting over several days suggests that potential mechanisms lie beyond mere extracellular ion concentration shifts or different time courses of relief from refractory ion channels. In fact, changes in the E/I ratio in response to variations in the input excitation to neuronal networks have been described in various in vitro and in vivo systems and are subsumed under the concept of *homeostatic plasticity*.^23^, ^24^ Thus, we hypothesize that extensive electrical perforant path stimulation could induce homeostatic plasticity processes such as synaptic scaling or adaptation of cellular excitability finally leading to a longer lasting shift in the local E/I ratio.^25^ The tight temporal association of the E/I reduction with the time point of perforant path‐mediated excitation of the dentate gyrus network (Figure 4B) suggests that the potentiation of feedforward or feedback inhibitory circuits are responsible for the observed effects. Although at this stage, discussion of possible specific plasticity mechanisms would be speculative, the sole enhancement of the excitability of GABAergic interneurons providing feedforward and feedback inhibition to granule cells could fully explain the observed epileptogenesis‐related alteration of the local E/I ratio. Indeed, for hippocampal parvalbumin‐positive (PV^+^) interneurons, different plasticity mechanisms leading to elevated excitability have been described: Intense synaptic excitation of PV+ interneurons in the dentate gyrus can enhance intrinsic excitability for prolonged periods of time by shifting the resting membrane potential to more depolarized levels.^26^ Furthermore, Donato and colleagues described different excitability states for PV^+^ interneurons with a highly excitable state corresponding to a high level of Parvalbumin and GAD67 expression being induced by strong excitation of the interneuron network.^27^ It is intriguing that alterations in the excitation of particularly PV^+^ interneurons have for decades been hypothesized to underlie the frequently observed disinhibition of dentate granule cells in different rodent epilepsy models and subsumed under the “dormant basket cell hypothesis.”^28^ Disinhibition of granule cells is reflected by the strong reduction in paired‐pulse inhibition assessed by extracellular field potential recordings upon perforant path stimulation.^28^, ^29^ Of interest, Sloviter and colleagues found with the same experimental protocol used in our study, after 30 min stimulations on Days 1 and 2, an increase in paired‐pulse inhibition corresponding well to the elevated aperiodic exponent identified at the corresponding time points (Figure 2D,E) and to the tendency toward a lower paired‐pulse index at the beginning of stimulation on Days 2 and 3 (Figure S3C). However, 1 day after completion of the epileptogenic insult, paired‐pulse inhibition is strongly reduced in many different epilepsy models^29^, ^30^ as it is at the end of each stimulation session in our own data set (Figure S3C). At the same time, our data indicate an initial transient period of elevated GABAergic synaptic activity shortly after the trauma before returning to baseline conditions over a few days. One explanation to reconcile these seemingly contradicting observations could be higher activity rates of GABAergic interneurons with yet impaired inhibitory efficiency on their postsynaptic targets, for example, by changes in the GABA_A_ receptor reversal potential—a phenomenon frequently detected in the context of epileptic circuits.^31^, ^32^

Whether the observed transient relative dominance of inhibitory tone is protective for the hippocampal circuits, as it could be a priori assumed, or alternatively contributes to epileptogenesis itself, needs to be further clarified. One argument in favor of a causal role of early inhibitory dominance for epilepsy development lies in the exclusive observation of the transient inhibition increase in *epilepsy* animals. A presumable protective mechanism could be expected to be more strongly expressed in those animals undergoing the potentially epileptogenic incident but remaining resilient against it. In agreement with this line of argumentation, maladaptive homeostatic plasticity has already been discussed as potential driving factors for ictogenesis and epileptogenesis.^33^, ^34^, ^35^ In fact, recently, patients with temporal lobe epilepsy were shown to express higher aperiodic exponents in seizure‐generating networks than their healthy counterparts.^36^ Mechanistically, a delayed, GABA‐triggered and microglia‐mediated elimination of GABAergic synapses on principal cells has been identified recently in the hippocampus during kainic acid–induced epileptogenesis and hypothesized to be one mechanism of established hyperexcitability mediated by an increased E/I ratio at later stages.^37^

Post‐insult transient changes in the E/I ratio may not only play a causative role in the generation and progression of epilepsy, but could, in principle, also underlie functional deficits in epileptic brain circuits. The impairment of brain network function in patients with chronic epilepsy is one of the dominant symptoms besides seizures.^38^ In our dataset, theta oscillations become less regular with progressing epileptogenesis (Figure 3D,E). As theta oscillations, particularly in rodents, are well known to serve as a reference signal for hippocampal information processing, the identified destruction of theta regularity might plausibly contribute to hippocampal dysfunction. Changes in the E/I ratio with elevated inhibition have been shown to, in general, affect brain theta oscillations in a similar way (reduced theta frequency and power)^39^ and to, more specifically, impair hippocampal theta‐dependent neuronal coding.^40^ However, the persistence of theta disruption outlasting the transient phase of increased aperiodic exponent suggests that theta disruption is not only a consequence of altered hippocampal E/I ratio. This is further supported by the fact, that in the dentate gyrus theta oscillations appear to be mostly propagated to the circuit via entorhinal input.^22^ Therefore, theta disruption indicates additional upstream network changes in the input areas of the dentate gyrus contributing to theta oscillation impairment.

What determines whether animals receiving the 3 day stimulation protocol are turning toward epilepsy or remain non‐epilepsy animals throughout? The easiest explanation would be inefficient stimulation of the perforant path. However, changes in the E/I ratio upon stimulation Days 1 and 2 were not different between *non‐epilepsy* and *epilepsy* animals (Figure 2D,E), suggesting an equally effective stimulation in both groups of animals. Furthermore, in several *non‐epilepsy* animals we recorded acute symptomatic seizures within the first 7 days after the electrical stimulation without detecting spontaneous seizures correlating with chronic epilepsy (Figure S1). Finally, our quantitative analysis of field responses to the perforant path stimulus did not detect significant differences in fEPSP amplitude and slope between epilepsy and non‐epilepsy animals (Figure S3). This illustrates clearly that, first, the transient post‐incidental increase in network inhibition correlates with chronic but not acute seizures and, second, the absence of changes in network inhibition—and very likely chronic epilepsy—is not due to insufficient stimulation. Thus, a fraction of animals seems to carry an inherent resilience against the electrical perforant path stimulation. This resilience may lie in the specific structure or activity state of the hippocampal network at the time point of stimulation. Alternatively, differences in the immunological response to the epileptogenic insult could underlie a resilience response to perforant path stimulation. Finally, a potential explanation emerges from the specific outline of the stimulation protocol. The 30 min‐long stimulation sessions on Days 1 and 2 of the stimulation protocol are designed as a preconditioning treatment in order to reduce the fraction of animals dying from the long‐lasting stimulation session on Day 3.^14^ The mechanisms bringing about tissue tolerance against the harmful electrical incident are currently unknown. However, similar preconditioning effects have been observed in different experimental epilepsy models, and diverse mechanisms have been suggested to underlie this so‐called *epileptic tolerance*.^41^ Thus, differential degrees of preconditioning elicited by stimulation Days 1 and 2 could in theory explain the variable responses to the third stimulation over 8 h. Future research investigating both, the processes underlying the development of epileptic tolerance but also the mechanisms of a potential resilience against epileptogenic incidents in the non‐epilepsy animals could open new opportunities for the development of an anti‐epileptogenesis treatment.

### 4.1 Technical limitations

One of the most evident points of criticism lies in the estimation of the ratio of inhibitory and excitatory synaptic activity by analyzing the aperiodic component of LFP power spectra. Since the distribution of this method by Voytek and colleagues,^11^ many neuroscientists have applied this easy‐to‐use methodology to address diverse highly relevant scientific questions. Although valid criticism has been brought forward including the recently detected intrusion of cardiac signals in aperiodic activity recorded from the brain,^42^ many example situations of evident shifts in the E/I ratio are paralleled by changes in the aperiodic exponent of the power spectra of respective neuronal signals.^11^, ^12^, ^39^, ^43^, ^44^, ^45^ Thus, although the analysis of aperiodic activity remains an indirect proxy for the actual ratio of excitatory and inhibitory synaptic activity in brain circuits, many lines of evidence indicate its usefulness in gross E/I ratio estimation. Indeed, our reproduction of the theta‐phase dependent rise in excitation shortly before the theta trough stands in very good agreement with published data^22^ and further confirms the validity of our E/I ratio‐estimation by aperiodic network activity analysis. Nevertheless, the exact cellular mechanisms underlying the changes in hippocampal network activity during early epileptogenesis need to be investigated in much more detail in future studies. Moreover, future research needs to assess whether a transient phase of inhibitory dominance is a general phenomenon of early epileptogenesis comprising both animal epilepsy models of different etiology and human epilepsy development.

## 5 CONCLUSION

In an electrically induced temporal lobe epilepsy model in rats, the process of epileptogenesis hidden to date can be electrophysiologically revealed as early as on the first day after the application of the epileptogenic stimulus by determining the time course of the aperiodic exponent of the hippocampal LFP. This finding has several potential implications for our understanding of epileptogenesis. First, network activity in epileptogenic circuits is drastically altered already 1 day after the incident.^29^ Second, synaptic inhibition might not only act protective during the process of epileptogenesis but may even drive the development of epileptic circuits at least during the initial days. Finally, these results provide an opportunity to dissect the mechanisms of protection from epileptogenesis by identifying future epileptic animals already immediately after stimulus application with a very high fidelity—questions inevitably related to the long‐held hope for an anti‐epileptogenesis treatment in high‐risk patients with potentially epileptogenic conditions such as intracranial trauma or new‐onset status epilepticus.

## AUTHOR CONTRIBUTIONS

M.S., T.M.S., R.K., J.T., A.S., and F.R. contributed to the conception and design of the study. M.S., T.M.S., V.S., L.C., V.N., P.G., J.P., K.S., and S.B. collected and analyzed the data. M.S., T.M.S., N.M., and R.K. wrote the manuscript. All authors revised and approved the final version of the manuscript.

## FUNDING INFORMATION

This work was supported by the LOEWE Center for PersonalizedTranslational Epilepsy Research (CePTER; Grant/Award Number: IIIL6‐ 519/03/03.001‐ (0007)) and by the ERDERA consortium on T‐cell‐based Approaches for Controlling Autoimmune Disease with Epilepsy (T‐CARE; DFG Project ID 583034287).

## CONFLICT OF INTEREST STATEMENT

A.S. has received personal fees and grants from Angelini Pharma, Biocodex, Desitin Arzneimittel, Eisai, Jazz Pharmaceuticals, Takeda, UCB Pharma, and UNEEG Medical. N.M. has received honoraria for lecturing and travel expenses for attending meetings from Biogen Idec, GlaxoSmith Kline, Teva, Novartis Pharma, Bayer Healthcare, Genzyme, Alexion Pharmaceuticals, Fresenius Medical Care, Diamed, UCB Pharma, AngeliniPharma, BIAL, and Sanofi‐Aventis; has received royalties for consulting from UCB Pharma, Alexion Pharmaceuticals, and Sanofi; and has received financial research support from Euroimmun, Fresenius Medical Care, Diamed, Alexion Pharmaceuticals, Novartis Pharma, and Sanofi. F.R. has received honoraria for lecturing and consultation from Angelini Pharma, Eisai GmbH, Jazz Pharma, Roche Pharma, Stoke Therapeutics, Takeda, and UCB Pharma; and has received financial research support from Dr. Schär Deutschland GmbH, Vitaflo Deutschland GmbH, Nutricia Milupa GmbH, Desitin Pharma, Hamburg, Federal State of Hessen (via the LOEWE‐Programme), Chaja Foundation Frankfurt, Reiss Foundation Frankfurt, Dr. Senckenbergische Foundation Frankfurt, Ernst Max von Grunelius Foundation Frankfurt, and Detlev‐Wrobel Fonds for Epilepsy Research Frankfurt, outside the submitted work. T.M.S. is a co‐founder and shareholder of Circulant GmbH. The remaining authors have no conflicts of interest.

## ETHICAL PUBLICATION STATEMENT

We confirm that we have read the Journal's position on issues involved in ethical publication and affirm that this report is consistent with those guidelines.

## SOCIAL MEDIA AND ARTICLE PROMOTION

This study shows the early detection of epileptogenesis by a reduced E/I ratio in an electrically induced temporal lobe epilepsy model.

## Supporting information


Figure S1.
Figure S2.
Figure S3.
Figure S4.
Data S1.

## Data Availability

The intracranial EEG data underlying the analysis of the aperiodic exponent are publicly available in our open access data repository at Zenodo (doi:10.5281/zenodo.18315373). Further data can be requested directly from the authors.
